# Posterior lumbar fusion surgery doesn’t change sexual activities in patients with lumbar degenerative disease: an observational study

**DOI:** 10.1186/s12891-023-06855-3

**Published:** 2023-09-12

**Authors:** Yukitoshi Shimamura, Masahiro Kanayama, Michiko Horio, Ai Yamaguchi, Fumihiro Oha, Takeru Tsujimoto, Masaru Tanaka, Yuichi Hasegawa, Tsutomu Endo, Tomoyuki Hashimoto

**Affiliations:** Spine Center, Hakodate Central General Hospital, Hon-Cho 33-2, Hakodate, Hokkaido 040-8585 Japan

**Keywords:** Posterior lumbar fusion surgery, Lumbar degenerative disease, Sexual activity

## Abstract

**Background:**

There are few studies about sexual function in the patient with posterior lumbar spinal fusion for degenerative lumbar disease. The aim of this study is to investigate sexual activities in patients with lumbar degenerative disease before and after lumbar fusion surgery.

**Methods:**

We recruited 35 patients who underwent lumbar spinal fusion at the age of 55 years or younger. They were 17 men and 18 women with a mean age of 47.4 years. After informed consent, the patients were asked to complete anonymous questionnaire concerning sexual desire, activity, and satisfaction before and after surgery.

**Results:**

In the presick period, 69% of the patients had sexual desire, and 79% achieved satisfaction during sexual activity. Lumbar degenerative disease decreased sexual desire and frequency of sexual activity in 40%, and 74% respectively. Before surgery, satisfaction in sexual activities decreased in 53%, and 55% of the patients felt discomfort during sexual activity. Adjustment in sexual position was required in 44% of man and 54% of woman. After surgery, Sexual desire, frequency of sexual activity and satisfaction did not regain after surgery in 94%, 93% and 92%, respectively.

Those who did not feel discomfort after surgery was significantly lower VAS in both low back pain and leg pain than the patients felt discomfort (low back pain; *p* = 0.024, leg pain; *p* = 0.046).

**Conclusion:**

This study demonstrated that lumbar degenerative diseases decreased sexual desire, frequency of sexual activity and satisfaction, and little of the patients regained their sexual activities after posterior lumbar fusion surgery in the middle-aged patients.

## Background

Sexual activities are important for quality of life (QOL) in many individuals. Since lumbar nerves are associated with sexual function, spinal pathology arising from trauma, deformity, and degenerative disease sometimes lead to sexual dysfunction [1–5]. Furthermore, lumbar spinal surgery might affect sexual activities according to postoperative mechanical, neurologic, and psychological factors. Regaining sexual function have a major impact on postoperative satisfaction.

Posterior lumbar fusion has become a common surgery for lumbar degenerative diseases. Fusion surgery provides pain relief by immobilizing painful motion segments and excluding nerve compression, improve sexual dysfunction occurred by low back pain and sciatic pain. Previous studies have evaluated about the relationship between sexual activities and low back pain, and some have investigated sexual function in patients who performed spinal surgery [1–3, 6–10]. However, it remains unclear whether or not fusion surgery regains sexual activities.

The aim of this study was to reveal the quality and frequency of sexual activities (sexual desire, activities, adjustment, and satisfaction) in patients with lumbar degenerative disease before and after surgery through a privacy-conscious anonymous survey.

### Patients and methods

A retrospective survey was performed for consecutive patients who underwent posterior lumbar fusion surgery for degenerative disease between January 2011 and September 2016. Exclusion criteria was as follows; 1) older than 55 years at the surgery, 2) surgery for trauma or tumor, and 3) disease affecting preoperative sexual function. Approval from our institutional review board was obtained prior to performing the study. We explained the purpose of this study at first for targeted patients, and 35 patients participated in this study with informed consent.

The survey was undertaken in a privacy-conscious fashion. Patients by themselves were asked to completed anonymous questionnaires in a separate room. We used published questionnaire regarding sexual desire, frequency, adjustment, and satisfaction before and after surgery (Table 1) [6]. They also filled out age, gender, marital state, duration of symptom, and presick status of sexual activity (sexual desire, frequency of sexual intercourse, and satisfaction). Visual analog scale (VAS) of low back pain and leg pain, changes of sexual activities before and after surgery were also recorded. After filling out the questionnaires, they dropped them into a sealed box. When all the patients dropped their questionnaires, the sealed box was opened for analysis. Although it is difficult to guarantee the accuracy of the sample information in detail because the questions for sexual activities is delicate, this method secured the accuracy of the sample information in some extent.

**Table 1:** Questionnaires concerning sexual activities

Before you had symptoms due to lumbar degenerative disease.	
Q1. Did you have sexual desire before the episode of lumbar degenerative disease?	Yes / No
Q2. How often did you have sexual intercourse?	( ) times/month
Q3. Did you have organism during sexual activities?	Yes / No
While you suffered from symptoms due to lumbar degenerative disease	
Q4. Have you experienced decreased sexual desire?	Yes / No
Q5. Did frequency of sexual intercourse decrease?	Yes / No
Q6. Have you experienced decreased ability to have organism?	Yes / No
Q7. Have you experienced discomfort during sexual activities?	Yes / No
Q8. Did you need adjustment in sexual position?	Yes / No
Q9. What was the most comfortable sexual position?	Missionary position (man on top of woman, face to face)
	Amazon position (woman on top of man, face to face)
	Spoons position (man from behind woman both lying on their side)
	Dog position (man from behind, woman on hands and knees)
	Lotus position (man sits cross-legged, woman sits on man)
Q10. What was the most painful sexual position?	Missionary/Amazon/Spoons/Dog/Lotus
After you underwent posterior lumbar fusion surgery	
Q11. Did you regain sexual desire after surgery?	Yes / No
Q12. Did frequency of sexual intercourse change?	Increased / unchanged / decreased
Q13. Did your ability to have organism change?	Increased / unchanged / decreased
Q14. When did you resume sexual activities after surgery?	( ) weeks after surgery

### Statistics

The results were analyzed using IBM SPSS Statistics for Windows (Version 20, IBM Corp.). Status of sexual desire, frequency, adjustment and satisfaction were statistically compared between man and woman using Chi-square test. Pre- and postoperative VAS data were compared using paired t-test. A *P* value < 0.05 was considered significant.

## Results

There were 17 men and 18 women with a mean age of 47.4 years (34—55 years) (Table 2).

**Table 2:** Characteristics of the patients

Variables	Total patients (*n* = 35)
Gender (man/woman)	17/18
Age at the surgery	47.4 (34–55)
Duration of symptom (month)	24.6 (1–240)
Diagnosis for surgery	
Degenerative spondylolisthesis	17
Isthmic spondylolisthesis	8
Lumbar foraminal stenosis	4
Lumbar canal stenosis	2
Lumbar disc herniation	1
Degenerative scoliosis	1
Relapsed lumbar disc herniation	1
Surgical method	
PLIF	19
TLIF	15
PF	1
Fusion segment	
One segment	29
Two segments	6

*PLIF* posterior lumbar interbody fusion, *TLIF* transforaminal lumbar interbody fusion, *PF* posterior fusion

Duration of symptom averaged 24.6 months. The diagnosis for surgery was degenerative spondylolisthesis in 17 cases, isthmic spondylolisthesis in 8, lumbar foraminal stenosis in 4, lumbar canal stenosis in 2, lumbar disc herniation, degenerative scoliosis, degenerative disc disease and recurrent lumbar disc herniation in one case each. The surgical method was posterior lumbar interbody fusion (PLIF) in 19 cases, transforaminal lumbar interbody fusion (TLIF) in 15, and posterior fusion (PF) in one. The number of fused segment was one segment in 29 cases, and two segments in six.

Of the 35 patients, 24 patients (69%) had sexual desire, and 26 patients (79%) achieved satisfaction during sexual activity in the presick periods. Frequency of sexual intercourse was averaged to 2.2 times/month (1.9 times in man, 2.5 times in woman). Proportion of those who had sexual desire was significantly higher in man than woman (man vs woman; 89% vs 50%, *P* = 0.015). There was no significant difference in gender regarding frequency of sexual intercourse (*P* = 0.394) and satisfaction (82% vs 75%, *P* = 0.606).

After the onset of disease, sexual activities were largely impaired. Sexual desire, satisfaction in sexual activity, frequency of sexual activities decreased in 40%, 53%, 74% of the patients, respectively. Also, 55% of patients felt discomfort during sexual activity. Gender difference was not significant in sexual desire (35% vs 44%, *P* = 0.581), frequency (71% vs 76% *P* = 0.697), satisfaction (50% vs 56%, *P* = 0.723), and discomfort (50% vs 60%, *P* = 0.576).

**Fig. 1:**
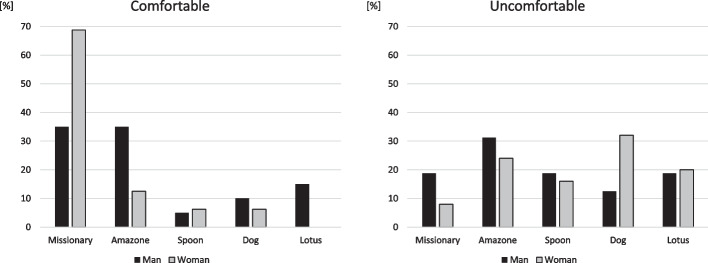
Comfortable or uncomfortable sexual position in patient with degenerative lumbar diseases before surgery

Adjustment in sexual position was required in 44% of men and 53% of women. There was also no significant difference in gender (*P* = 0.594). The most comfortable position was missionary position or amazon position in men, and missionary position in women. The most uncomfortable position was dog position or amazon position in women. The most uncomfortable position in men divided into each one of the position (Fig. 1).

VAS (0 – 100) of low back pain and sciatic pain were 66.6 ± 35.4 and 63.8 ± 36.1 before surgery, and significantly improved to 16.6 ± 22.0 and 13.2 ± 25.3 after surgery, respectively (*P* < 0.001).

**Fig. 2:**
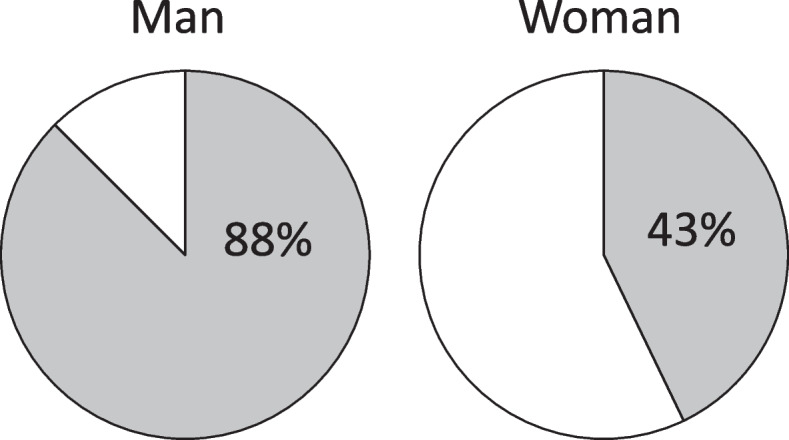
Patients who feel discomfort during sexual activity after surgery

Sexual desire, frequency of sexual activity and satisfaction did not regain after surgery in 94%, 93% and 92% of the patients, respectively. The duration to resume sexual activity was 11.6 weeks in men and 16.9 weeks in women. Men tended to resume sexual activities earlier than women (*P* = 0.376). Also, 67% of those who had felt discomfort during sexual activity before surgery also felt discomfort after surgery, and men tended to feel discomfort compared to women (88% vs 43%, *P* = 0.067) (Fig. 2). Those who did not feel discomfort after surgery had significantly lower VAS of low back pain and leg pain than those who felt discomfort (low back pain; *P* = 0.024, leg pain; *P* = 0.046) (Table 3).

**Table 3:** Comparison of pre- and post-operative VAS between those with and without feeling discomfort

	discomfort	no discomfort	*p*
Preoperative VAS			
Lumbar pain	84.1 ± 21.8	58.8 ± 36.7	0.133
Leg pain	61.0 ± 34.4	78.2 ± 29.4	0.325
Postoperative VAS			
Lumbar pain	34.2 ± 30.5	7.4 ± 7.3	0.024
Leg pain	30.7 ± 38.8	2.2 ± 2.3	0.046

*VAS* Visual analog scale

## Discussion

Both clinicians and patients tend to be avoid discussing sexual activities in several reasons such as old age of patients, lack of knowledge, lack of patient’s initiative, and shame of bring up the subject. Therefore, the evaluation about how sexual activities change before and after surgery often be ignored in most cases [11].

Sexual function after anterior lumbar interbody fusion (ALIF) have been reported because retrograde ejaculation was predisposed to occur after ALIF by damage to the hypogastric nerve plexus [12, 13]. However, there were few studies that reported sexual desire and activities in patients performed posterior fusion surgery for lumbar degenerative diseases. This study revealed the quality and frequency of sexual activities in patients with lumbar degenerative diseases before and after posterior lumbar fusion surgery through a privacy-conscious anonymous survey.

This study was conducted targeting the middle-aged patients; a mean age was 47.4 years. Sexual activity might be less frequently in the middle-aged group than the young group. However this study clarified 69% of patients had sexual desire, 79% of patients achieved satisfaction during sexual activity and frequency of sexual intercourse was averaged to 2.2 times/month in presick periods. These results suggested postoperative sexual activities are not minor problems in the middle-aged patients.

Lumbar degenerative diseases decreased sexual desires, frequency of sexual activity and satisfaction compared to presick period, and required adjustment of sexual position. Also, Horst et al. reported that sex life was relevant to the majority of patients with lumbar degenerative diseases and 55% of these had some pain affecting their sex life [1]. As lumbar degenerative diseases affect sexual activities for high rate of patients, the physicians should pay attention to these problems.

Several studies examined the effect of surgical intervention on the sexual activities. Berg et al. investigated sexual life in 152 patients with symptomatic degenerative disc disease who underwent total disc replacement or PLF/PLIF. They reported that sex life improved after surgery [8]. While on the other hand, Hagg et al. reported sexual enjoyment at 2-years follow-up in 169 patients who underwent PF or ALIF for chronic low back pain, and it remained unchanged or got worse in 62% of patients after surgery [7]. The current study demonstrated that sexual activities after surgery remained unchanged or got worse compared with before surgery in most of the patients. This suggested that posterior lumbar fusion surgery has a limitation to improve the decreased sexual desire, frequency, and satisfaction.

As well, those who felt discomfort during sexual activities after surgery had higher VAS of low back pain and leg pain. Previous studies have evaluated about the relationship between sexual activity and low back pain. Nikoobakht et al. reported that chronic low back pain patients report considerably higher prevalence of sexual problems compared with healthy controls [4]. Berg et al. explored 152 patients with low back pain, reported that 84% of the patients retained difficulty in their sex activities, and improvement of sex life was strongly correlate to a reduction of low back pain [8]. Sexual activity after surgery could be greatly affected by improvement of low back pain and leg pain.

Decreasing of sexual activities might be occurred by not only the damage of cauda equina but also low back pain and leg pain. Kanayama et al. reported surgery for lumbar disc herniation improved sexual desire, frequency of sexual activity and satisfaction. This result suggested pain relief also improves sexual activities. However, sexual activities didn’t improve well in present study, despite including lumbar spondylolisthesis and foraminal stenosis which presents with leg pain as the chief complaint. We speculate the reason for difference from the previous study is as followed; 1) the duration of degenerative lumbar disease is relatively long, so once sexual desire decreased for a long period, sexual activities don’t improve easily after surgery in the middle-aged patients. 2) postoperative VAS back pain was higher in lumbar fusion surgery than lumbar disc herniation surgery.

There were limitations in this study. First, this study was a retrospective study, and the results rely on long-term recall of presick, preoperative and postoperative sexual activities. Second, we could not track the individual clinical and radiographic data because of anonymous survey. As questionnaires related to sexual activities included sensitive issues, we should particularly pay attention to privacy-conscious fashion to obtain reliable answer. Third, information of all the drugs they took wasn’t investigated in this study. There might be patients who have taken the drug affecting their sexual activities during perioperative period, Finally, this study included small sample size might not be enough to clarify the association between sexual activities and lumbar fusion surgery in detail. Indeed, sexual activities were affected by not only patient factors but also partner-related factors [14]. Further large sample size study is needed to completely elucidate these points.

## Conclusion

This study demonstrated that lumbar degenerative diseases decreased sexual desire, frequency of sexual activities and satisfaction, and little of the patients regained their sexual activity after posterior lumbar fusion surgery in the middle-aged patients.

## Data Availability

All data included in this study are available upon requests by contact with the corresponding author on reasonable request.
